# Immediate Placement and Restoration of a New Tapered Implant System in the Aesthetic Region: A Report of Three Cases

**DOI:** 10.1155/2020/7632692

**Published:** 2020-07-25

**Authors:** Caroliene M. Meijndert, Gerry M. Raghoebar, Arjan Vissink, Henny J. A. Meijer

**Affiliations:** ^1^Department of Oral and Maxillofacial Surgery, University of Groningen, University Medical Center Groningen, Groningen, Netherlands; ^2^Department of Implant Dentistry, Dental School, University of Groningen, University Medical Center Groningen, Groningen, Netherlands

## Abstract

**Objective:**

To assess the clinical, radiographic, aesthetic, and patient-centred outcomes of a new implant system applied for an immediate implant placement and restoration approach in single tooth replacement of anterior maxillary teeth. *Material and Method*. Three cases were treated with a bone level tapered implant. All patients were treated with the same strategy involving flapless extraction and implant placement with simultaneous augmentation. Implants were provisionally restored with a screw-retained restoration at the day of surgery. Definitive restoration was fabricated after 3 months. Follow-up was one year after definitive restoration.

**Results:**

At the 1-year follow-up, the implants were stable and no complications had occurred. Peri-implant bone levels had increased with a mean value of 0.24 ± 0.30 mm between definitive restoration placement and 1 year of follow-up. Clinical outcome scores showed healthy soft tissues. Mean Pink and White Esthetic scores were rated 7.0 and 7.3, respectively. Mean patient satisfaction had improved from 55.7 (pretreatment) to 90.0 (1-year follow-up) on a 0-100 VAS scale.

**Conclusion:**

Immediate implant placement and restoration with the new tapered bone level implant system are accompanied by good initial clinical and radiographic results as well as high patient satisfaction.

## 1. Introduction

Implant placement and restorative procedures have evolved into a procedure that can be performed immediately after tooth extraction. This approach is less time-consuming than the conventional procedure and leads to increasing patient contentment [1]. This is especially beneficial in an aesthetically sensitive area like the anterior maxilla. Studies have shown that this procedure has an outcome comparable to the conventional implant placement and restoration protocols [2, 3].

One of the key conditions for the success of an immediate implant placement and restoration approach is primary stability [4]. Primary stability limits micro movement and allows osteogenic cells to adhere to the implant surface, leading to osseointegration [5, 6]. Factors that influence primary stability are the quality and quantity of bone, surgical techniques, and implant design [7]. If osseointegration is achieved, the next challenge is to maintain stable peri-implant hard and soft tissues. This too has been a topic of research. Amongst others, to reduce peri-implant bone resorption, improvements have been made at the implant-abutment connection by platform switching and by using an internal connection type [8]. An internal conical connection is presumed to be accompanied by less peri-implant bone loss compared to either an external connection or a straight internal connection [9, 10].

A new implant line has recently been presented by Straumann. The bone level implant is equipped with a self-cutting thread design and internal conical implant-abutment connection for high primary stability and minimal bone loss in fresh extraction sockets.

The aim of this case series study is to describe three cases with a failing tooth in the aesthetic region (p1-p1) that were treated with this implant system following an immediate implant placement and restoration protocol.

## 2. Material and Methods

### 2.1. Study Design

This report describes a prospective case series with a follow-up of one year. Recruitment of patients, implant treatment, and follow-up took place at the Department of Oral and Maxillofacial Surgery of the University Medical Centre Groningen, the Netherlands.

### 2.2. Patients

Patients were eligible if they met the inclusion criteria: >18year of age, adequate oral hygiene, nonsmoking, no medical and general contraindications for the surgical procedure (ASA score ≥ III [11]), and no periodontal pathology in the remaining dentition, indicated by bleeding on probing combined with pockets ≥ 4 mm. An intraoral radiograph and a cone beam computed tomography (CBCT) were made to determine whether it was likely to expect that the implant would gain sufficient initial stability immediately after tooth removal. Recommendations of Buser et al. [12] were followed concerning immediate implant placement: a fully intact facial bone wall at the extraction site with a thick wall phenotype (>1 mm), a thick gingival phenotype, no acute infection at the extraction site, and sufficient volume of bone apical and palatal of the extraction site to allow implant insertion in a correct 3D position with sufficient primary stability.

**Figure 1:**
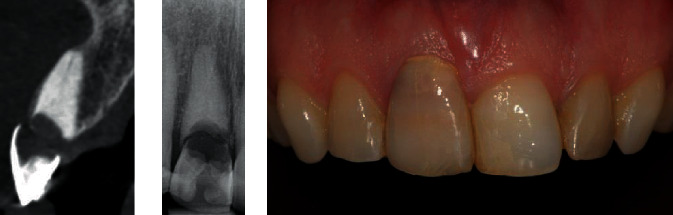
Initial situation before treatment of one of the included patients. Note the intact facial bone wall, having at least a thickness of 1 mm in the cervical region.

Three patients were included: two presented with an irreparable resorption defect in the right central incisor and one patient was referred for a replacement of a fractured left central incisor; (one case is presented to illustrate; see Figure 1). After detailed explanation of the benefits and risks of possible treatment options, the patients chose an implant-supported restoration to replace the failing central incisor. Written informed consents were obtained from the patients before enrolment. All patients were treated following the same immediate placement and restoration protocol as described below.

### 2.3. After Inclusion

After inclusion, an alginate impression was made and sent to the dental lab where a plaster model was fabricated. An open impression tray was printed based on a scanned version of the plaster cast, and a surgical drilling template was manually produced according to the ideal position of the future crown. The preferred implant position was planned so that the restoration could be screw retained.

### 2.4. Surgical Procedure

**Figure 2:**
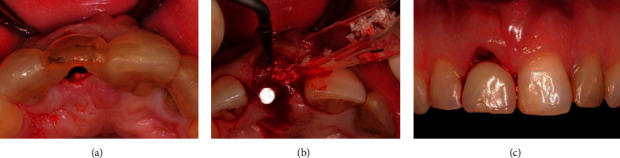
Surgical phase: (a) surgical template in place, (b) bone graft mixture added around the final drill, and (c) restoration at the same day as the implant placement.

**Figure 3:**
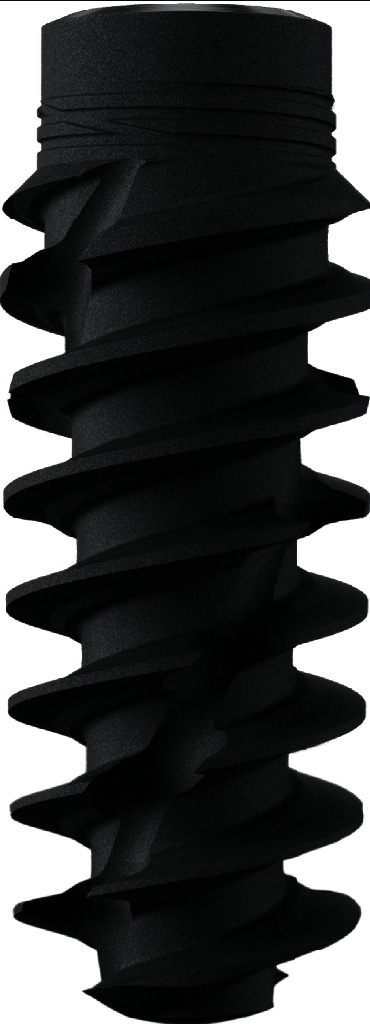
Straumann BLX implant RB. Roxolid® implant material with SLActive® surface. Full tapered core with self-cutting threads, chip flute for redistribution of bone chips, and microthreads on the implant neck to reduce stress at the cortical bone. Conical implant abutment connection (TorcFit™). Courtesy of Straumann AG.

Patients started prophylactic antibiotic treatment one day before the surgery (amoxicillin 500 mg, three times daily for 7 days) and twice a day used a 0.2% chlorhexidine mouth rinse (Corsodyl; GlaxoSmithKline, Utrecht, the Netherlands). After administering local anaesthesia (Ultracaine D-S Forte; Aventis Pharma Deutschland GmbH, Frankfurt am Main, Germany), the periodontal fibers were separated from the tooth after which the affected tooth could be carefully removed without raising a flap. The alveolus was carefully inspected and cleaned of residual granulation tissue. The manufacturer's drilling protocol was followed starting with the needle drill marking the site of implant insertion as dictated by the (semiguided) drilling template (Figure 2(a)). The surgeon ensured that the implant was positioned slightly palatal to the axis of the original root, for sufficient initial stability and ensuring a buccal bone thickness of at least 2 mm in the cervical region. The final implant drill was placed in the osteotomy as a space maintainer during augmentation of the gap between the drill and buccal wall. A 1 : 1 mixture of autologous bone (residual bone chips collected from the burs during osteotomy) and anorganic bovine bone (Geistlich Bio-Oss, Geistlich Pharma AG) was used (Figure 2(b)). Next, the final drill was carefully removed and the implant (Straumann BLX implant, Strauman AG, Basel, Switzerland (Figure 3)) was placed 3 mm apical to the most apical aspect of the prospective clinical crown. The implant was torqued to 45 Ncm. An implant-level open tray impression was made with a vinylpolysiloxane precision impression material (Provil Novo, Medium fast set. Kulzer Mitsui Chemical Group, Germany). The impression was sent to the dental lab for manufacturing the provisional restoration. A healing abutment was placed on the implant. A sterile reabsorbable gelatin sponge haemostat (Cutanplast®, Mascia Brunelli, Milan, Italy) was applied to seal the graft material from the time of placement of the healing abutment until the placement of the provisional restoration.

### 2.5. Prosthetic Procedure

**Figure 4:**
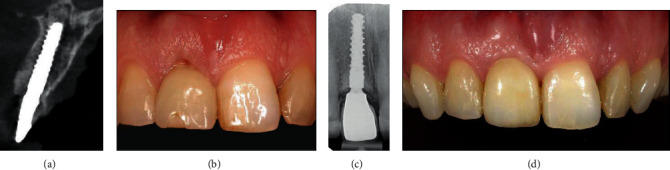
(a) Control CBCT at the day of implant placement. (b) Temporary crown 2 weeks after placement. (c, d) Radiographic and clinical images 12 months after definitive crown placement.

At the end of the same day, the healing abutment was replaced by the provisional restoration (Figure 2(c)). The provisional consisted of a screw-retained, platform switched titanium stock abutment with an acrylic resin crown. The crown was under-contoured to allow the gingiva to regrow for better aesthetics at final crown stage. The temporary crown was torqued to 25 Ncm and left to heal for 3 months. It was checked that there was no contact with antagonistic teeth during articulation. A CBCT was made to check the implant position (Figure 4(a)).

Two weeks after implant placement and temporary provisionalization, the patients were recalled for checkup (Figure 4(b)). None of the patients reported pain at the time of recall, and only one patient reported having a bruised feeling at the day of implant surgery and one patient reported to have lost a few granulation particles but no major dehiscence was present.

After a 3-month healing period, an open tray implant level impression was made with a polyether precision impression material (Impregum Penta; 3 M ESPE, St. Paul, USA) for the purpose of a definitive restoration. The definitive restoration consisted of a titanium base with zirconium structure and porcelain crown. When the restoration was completed and patients were satisfied, the crown was placed and tightened to 35 Ncm torque and oral hygiene instruction was given. Patients were followed for 12 months after final crown placement (Figures 4(c) and 4(d)).

### 2.6. Follow-Up

Recall was at 1 month (*T*_1_) and 12 months (*T*_12_) after the final restoration was placed. On these follow-up appointments, X-rays were made and clinical variables were measured. Bone level change was measured on a periapical radiograph on the mesial and distal sides of the implant by drawing a line from the implant shoulder to the bone to implant contact point. Change in the midbuccal gingiva level was measured by drawing a (horizontal) line from the top of the midbuccal zenith of both natural lateral incisors and a second (vertical) line was drawn, perpendicular from the horizontal line to the mid buccal zenith of the tooth/implant crown. The length of this vertical line was measured. A periodontal probe was held close to and parallel to the long axis of the implant crown and was used as a calibration [13].

Clinical variables, being the bleeding score (modified Sulcus Bleeding index [14]) and pocket probing depth (using the Clickprobe with standard pressure of 0.2-0.25 N of KerrHawe Dental Corporation, Bioggio, Switzerland) were scored. Patient satisfaction was scored on a 0-100 mm VAS-scale. Aesthetic outcome was rated on intra-oral photographs using the modified Pink and White Esthetic Score (PES-WES) [15].

## 3. Results

Results of the outcome measures are depicted in Table 1.

No biological or technical complications had occurred during the whole follow-up time, all implants were stable, and patients were very satisfied with the final result (survival rate 100%).

**Figure 5:**
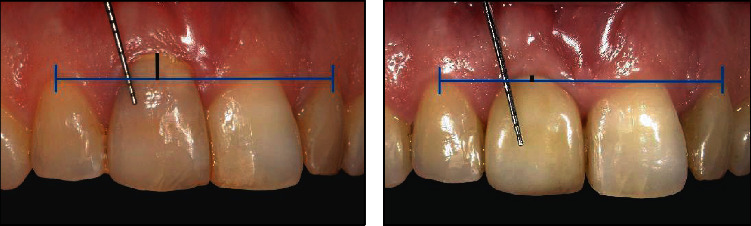
An improvement (1.74 mm) of midbuccal zenith after one year compared to the situation before treatment.

The peri-implant characteristics of the patient of case 1 started with a gross deviation in midbuccal zenith of 2.24 mm more apical compared to the contralateral incisor. This was improved at the *T*_12_ measurements, but a slight mismatch of 0.69 mm remained (Figure 5). This did not bother the patient, so no further treatment was initiated. Clinical assessment showed one isolated bleeding on probing at the distobuccal implant site. Pocket probing measured at this site was 3 mm. Radiographs showed high attachment of the bone to the implant up to the implant shoulder.

The peri-implant tissue of patient case 2 showed moderate peri-implant inflammation expressed by a confluent red line on probing, redness, and slight edema of the gingiva sulcus. Pockets were ≤4 mm. Inflammatory signs were also detected at the neighbouring teeth showed by bleeding on probing and slight redness and edema of the sulcular borders and pockets ≤ 3 mm. The patient was pregnant at the time of *T*_12_ follow-up.

The patient described in case 3 started with no crown to score for PES/WES; therefore, PES/WES was not rated prior to treatment. The patient was very satisfied with aesthetic and functional abilities at the 12-month follow-up. Bleeding on probing was not detected. The periapical radiograph showed good maturation of bone with minimal change in the bone level.

Overall, implant survival rate in the three patients was 100%. The mean bone level increased with 0.24 ± 0.30 mm. The mean PES score improved from score 4.0 at *T*_Pre_ to 7.0 at *T*_12_, WES improved from 3 at *T*_Pre_ to 7.3 at *T*_12_, patient satisfaction improved from 55.7 at *T*_Pre_ to 90.0 at *T*_12_ on the VAS scale. No technical or biological complications occurred up to 1-year follow up.

## 4. Discussion

This case series presents the results of three patients that were treated with the new Straumann BLX tapered bone level implant, immediately after tooth extraction.

After 15 months in function, both patients reported outcome and aesthetic scores were much improved compared to the pretreatment situation. The three patients presented with satisfying hard and soft peri-implant tissues.

Radiographic images 12 months after definitive crown placement (the implants were 15 months in function at this time) show a mean bone level change of +0.24 ± 0.30 mm. de Bruyn et al. reported a bone change of +1.3 mm after 1 year in an immediate implant placement group. They attributed this bone gain to healing of the extraction socket [16]. It is not unlikely that a similar mechanism applies here. The implant is also equipped with features that, in other studies, are shown to be favourable in the preservation of cortical bone around the implant, for example, a platform switch [17, 18] and a conical implant-abutment connection [10, 19–21]. There is not much known about the effect of the connection configuration on the aesthetic outcome in the anterior region. Cooper et al. compared three different connection configurations in the anterior region. They found the least bone loss in the conical connection group but found no statistical significant difference in the papilla level change or the PES score. They stated that more research on the effect of implant-abutment interface design is needed [20, 21].

The three studied patients in this report presented with satisfying improvements as expressed in a PES score of 7.0 and patient questionnaire (VAS-scale score of 90.0). This is in line with other studies on immediate placement and provisionalization protocols, where PES scores of 7.5 ± 1.6 [22] and 6.8 ± 1.5 [23] were published on immediate implant placement protocols. All patients reached the threshold of clinical acceptability (score ≥ 6) [15].

One patient was pregnant at the final follow-up and showed signs of inflammation around the implant and other teeth. There is a connection between increased plasma levels during pregnancy and gum disease [24]. Because the same inflammatory signs were present at the neighbouring teeth, it can be assumed that the marginal peri-implant soft tissues are affected due to the pregnancy hormones.

The initial results of this implant are favourable. However, this is a report of three patients followed for a short period of time. Studies with larger population and a longer follow-up are needed to view the stability of the facial hard and soft tissues in the long-term, as various studies have shown that hard and soft tissue alterations can be observed after many years in function [25–27].

## 5. Conclusion

Within the limitations of this study, it can be concluded that immediate implant placement and provisionalization using this implant have the potential to result in favourable clinical, radiographic, aesthetic, and patient satisfaction outcomes.

## Figures and Tables

**Table 1:** Clinical, radiographic, and patient-centred outcomes.

	Mesial bone level change (mm) *T*_1_-*T*_12_^∗^	Distal bone level change (mm) *T*_1_-*T*_12_^∗^	Mean midbuccal gingiva level change (mm) *T*_Pre_ to *T*_12_^**‡**^	Mean pockets at *T*_12_ (mm)	Bleeding score at *T*_12_	VAS-score patient satisfaction		PES		WES	
						*T*_Pre_	*T*_12_	*T*_Pre_	*T*_12_	*T*_Pre_	*T*_12_
Case 1	0.00	0.00	+1.74	2.3	1	58	92	4	8	3	7
Case 2	+0.30	0.00	+0.73	3.5	2	56	87	8	8	6	7
Case 3	+0.11	+1.05	+0.32	2.3	0	53	91	na	5	na	8
Mean	0.24 ± 0.15	0.35 ± 0.61	0.93 ± 0.73	2.7	1	55.7	90.0	6.0	7.0	4.5	7.3

*T*_Pre_ = situation before treatment; *T*_1_ = 1 month after definitive crown placement; *T*_12_ = 12 months after definitive crown placement; na = not applicable. ^∗^Positive value indicates that bone level was increased towards the implant shoulder. Zero indicates bone depicted at or above the implant shoulder at both *T*_1_ and *T*_12_. ^‡^Positive value indicates an incisal displacement of the mid buccal zenith.
